# Serum IL-6 and IL-23 Levels and Their Correlation with Angiogenic Cytokines and Disease Activity in Ankylosing Spondylitis, Psoriatic Arthritis, and SAPHO Syndrome

**DOI:** 10.1155/2015/785705

**Published:** 2015-08-03

**Authors:** Hanna Przepiera-Będzak, Katarzyna Fischer, Marek Brzosko

**Affiliations:** ^1^Department of Rheumatology and Internal Diseases, Pomeranian Medical University in Szczecin, Unii Lubelskiej 1, 71-252 Szczecin, Poland; ^2^Independent Laboratory of Rheumatic Diagnostics, Pomeranian Medical University in Szczecin, Unii Lubelskiej 1, 71-252 Szczecin, Poland

## Abstract

*Objectives.* To assess serum interleukin-6 (IL-6) and interleukin-23 (IL-23) and their correlation with angiogenic cytokines and disease activity in ankylosing spondylitis (AS), psoriatic arthritis (PsA), and SAPHO syndrome. *Patients and Methods.* We studied 152 spondyloarthritis (SpA) patients: 69 PsA, 61 AS, 22 SAPHO, and 29 controls. We recorded age, sex, disease duration, and treatment. We assessed BASDAI, VAS, and PASI scores. Serum IL-6, IL-23, VEGF, EGF, FGFb, and FGFa levels were determined using ELISA. We estimated ESR and CRP. *Results.* Serum IL-6 and IL-23 levels were higher in SpA than in control (*P* < 0.00001 and *P* = 0.0004, resp.). There was a positive correlation between serum IL-6 and CRP in AS (*P* = 0.000001), PsA (*P* = 0.000001), and SAPHO (*P* = 0.0003) patients. There was a positive correlation between serum IL-6 and ESR in AS (*P* = 0.000001), PsA (*P* = 0.002), and SAPHO (*P* = 0.02) patients. There was no correlation of serum IL-6 and IL-23 with VAS, BASDAI, and angiogenic cytokines in SpA. *Conclusions.* Serum IL-6 but not serum IL-23 correlated with ESR and CRP in SpA. No correlation was found of serum IL-6 and IL-23 with VAS, BASDAI, and angiogenic cytokines.

## 1. Introduction

Spondyloarthropathies (SpA) are a group of chronic, inflammatory, immune-mediated disorders of the axial and peripheral joints. This group encompasses ankylosing spondylitis (AS), psoriatic arthritis (PsA), reactive arthritis, enteropathic arthritis, and undifferentiated seronegative arthritis. SAPHO (synovitis, acne, pustulosis, hyperostosis, and osteitis) syndrome is always considered as a SpA. The aetiology of SpA is unknown, but some cytokines such as interleukin-6 (IL-6) and interleukin-23 (IL-23) are considered to be associated with the pathogenesis of these disorders [1–7].

IL-6 is a pleiotropic cytokine that plays role in arthritis but its role in the pathogenesis of AS remains controversial [1, 3]. IL-23 is produced by dendritic cells, macrophages, keratinocytes, and other antigen-presenting cells. There are some data showing that IL-23 plays an important role in the pathogenesis of spondyloarthritis [4]. In the available literature, we found that few reports assessed serum levels of IL-23 in small groups of AS and PsA patients [8, 9]. There were no data in the available literature concerning serum levels of IL-6 and IL-23 in SAPHO syndrome.

Angiogenesis also plays an important role in the pathogenesis of SpA. The group of cytokines involved in angiogenesis includes vascular endothelial growth factor (VEGF), epidermal growth factor (EGF), and basic and acidic fibroblast growth factors (FGFb and FGFa, resp.) [10–13].

There were no data available in the literature comparing serum levels of IL-6 and IL-23 and angiogenic cytokines in AS, PsA, and SAPHO.

## 2. Objectives

The aim of this study was to assess serum levels of IL-6 and IL-23 and their association with disease activity in AS, PsA, and SAPHO patients.

## 3. Materials and Methods

This study was approved by the Local Ethics Committee of Pomeranian Medical University in Szczecin. Informed consent was obtained from all patients.

All patients were Caucasian. We studied 152 patients: 69 had PsA, 61 had AS, and 22 had SAPHO. The controls were 29 healthy volunteers.

The diagnosis of AS was made according to modified New York criteria [14]. The diagnosis of PsA was made according to the Caspar classification criteria [15]. The diagnosis of SAPHO syndrome was made according to the Kahn criteria [16].

The following data were recorded: age, sex, disease duration, presence of peripheral or axial joint involvement, type of skin psoriasis, nail involvement, and treatment. In the PsA group, skin changes were assessed according to the Psoriasis Area and Severity Index (PASI) [17].

We assessed the Bath Ankylosing Spondylitis Disease Activity Index (BASDAI). This index has a possible score of 0–10, with a higher score indicating greater disease activity. We regarded patients as active if the BASDAI score was >4 [18]. The patient's pain due to the disease at the time of examination was assessed by a visual analogue scale (VAS).

Blood was taken for the assessment of ESR and C-reactive protein (CRP) (turbidimetric nephelometry, rate of reaction). Serum was stored at −70°C until analysis for IL-6, IL-23, VEGF, EGF, FGFb, and FGFa using a sensitive sandwich ELISA method using the Human IL-6 Immunoassay Quantikine ELISA kit (the minimum detectable dose less than 0.7 pg/mL), Human IL-23 Immunoassay Quantikine ELISA kit (the minimum detectable dose less than 6.8 pg/mL), Human VEGF Immunoassay Quantikine ELISA kit (the minimum detectable dose less than 5.0 pg/mL), Human EGF Immunoassay Quantikine ELISA kit (the minimum detectable dose less than 0.7 pg/mL), Human FGF Basic Immunoassay Quantikine ELISA kit (the minimum detectable dose less than 3 pg/mL), and Human FGF Acidic Immunoassay Quantikine ELISA kit (the minimum detectable dose less than 5.68 pg/mL). All kits were from R&D System, Minneapolis, USA. The system uses microplates with the walls coated with a monoclonal antibody and an enzyme-linked polyclonal antibody specific for IL-6, IL-23, VEGF, EGF, FGFb, or FGFa. All analyses and calibrations were performed in duplicate and were read using BioTek PowerWave XS, BioTek Instruments, Winooski, USA.

Data distributions were assessed using Shapiro-Wilk test. We used the rank Spearman test to calculate correlations. *R* values of correlations were determined and corresponding *P* values < 0.05 were considered significant. The groups were compared using Mann-Whitney *U* test and Kruskal-Wallis test. To assess parameters associated with serum levels of IL-6 and IL-23 a Pearson chi-squared test (*χ* ^2^), logistic regression analysis, and stepwise analysis were performed.

The level of significance was set at *P* < 0.05. Statistical analysis was performed using STATISTICA version 6.0.

## 4. Results

The clinical and laboratory characteristics of the patients and healthy controls are presented in Table 1.

In the AS group, 24 patients received nonsteroidal anti-inflammatory drugs (NSAIDs), 26 received sulfasalazine 2 g/day, and 11 received methotrexate 15 mg/week. In the PsA group, 17 patients received methotrexate 15 mg/day, 2 received methotrexate 15 mg/week in combination with cyclosporine A 3 mg/kg, 35 received sulfasalazine 2 g/day, and 15 received NSAIDs. In the SAPHO group, 10 received methotrexate 15 mg/week, 8 received sulfasalazine 1 g/day, and 4 received NSAIDs. No patients received biological therapy.

**Figure 1:**
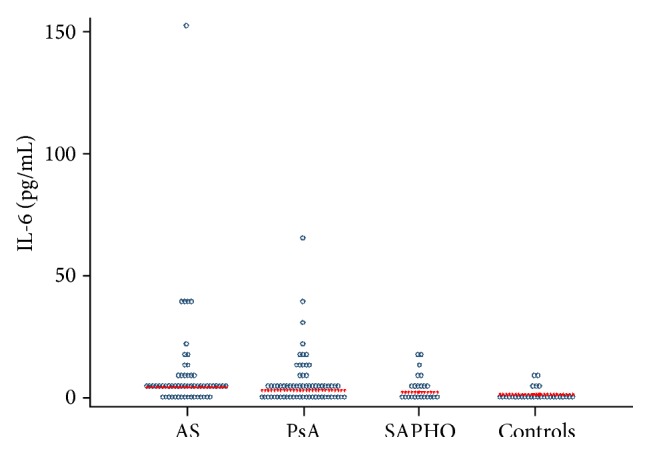
Serum levels of interleukin-6 (IL-6) in patients with psoriatic arthritis (PsA), ankylosing spondylitis (AS), and SAPHO syndrome (SAPHO) and controls.

Serum IL-6 levels were significantly higher in SpA patients than in the control group (*P* < 0.00001). Serum IL-6 levels were significantly higher in AS than in SAPHO patients (*P* = 0.04). No differences were found between AS and PsA patients (*P* = 0.21) or between PsA and SAPHO patients (*P* = 0.27) in terms of IL-6 levels (Figure 1).

**Figure 2:**
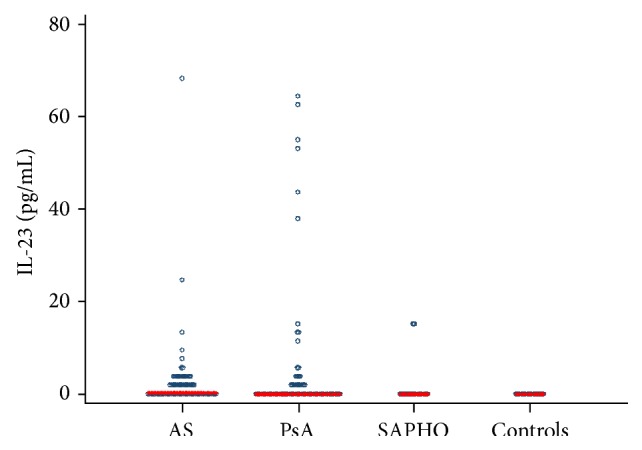
Serum levels of interleukin-23 (IL-23) in patients with psoriatic arthritis (PsA), ankylosing spondylitis (AS), and SAPHO syndrome (SAPHO) and controls.

Serum IL-23 levels were significantly higher in SpA patients than in the control group (*P* = 0.0004). Serum IL-23 levels were significantly higher in AS than in SAPHO patients (*P* = 0.03). No differences were found between AS and PsA patients (*P* = 0.17) or between PsA and SAPHO patients (*P* = 0.21) in terms of IL-6 levels (Figure 2).

Serum VEGF, EGF, FGFb, and FGFa levels were similar in AS, PsA, and SAPHO patients and controls (Kruskal-Wallis test: *P* > 0.05).

All PsA patients had plaque-type psoriasis. IL-23 and IL-6 levels were not correlated with PASI score (Tables 2 and 3). Among PsA patients, 57 (82.6%) had peripheral arthritis (31 had polyarthritis, 20 had oligoarthritis, and 6 had distal arthritis) and 12 (17.4%) had axial disease. No differences were found between patients with different forms of PsA presentation in terms of IL-6 levels (Kruskal-Wallis test: *P* > 0.05) and IL-23 levels (Kruskal-Wallis test: *P* > 0.05).

Among AS patients, peripheral arthritis was present in 23 (37.7%). No differences were found between patients with different forms of AS presentation in terms of IL-6 levels (*P* > 0.05) and IL-23 levels (*P* > 0.05).

There was a positive correlation between serum IL-6 and CRP in AS (*P* = 0.000001), PsA (*P* = 0.000001), and SAPHO (*P* = 0.0003) patients (data not shown). There was a positive correlation between serum IL-6 and ESR in AS (*P* = 0.000001), PsA (*P* = 0.002), and SAPHO (*P* = 0.02) patients (data not shown).

There was no correlation between IL-6, VAS, BASDAI, and angiogenic cytokines (VEGF, EGF, FGFb, and FGFa) (*P* > 0.05) in SpA patients (data not shown). There was no correlation between serum IL-23, CRP, ESR, VAS, BASDAI, and angiogenic cytokines (*P* > 0.05) in SpA patients (data not shown).

No differences were found between SpA groups of various treatment regimens in terms of IL-6 (Kruskal-Wallis test: *P* > 0.05) and IL-23 levels (Kruskal-Wallis test: *P* > 0.05).

The results of univariable and multivariable logistic regression analysis and stepwise analysis of serum IL-6 levels in SpA patients with adjustment to CRP, BASDAI, and DMARDs showed no association with BASDAI and treatment with DMARDs. The adjusted OR for serum IL-6 ≥ 1.53 pg/mL in SpA patients with increased CRP level (≥5 mg/L) was 7.68 (95% CI 2.93–20.16), *P* < 0.0001 (Table 2). In SpA patients with increased CRP level (≥5 mg/L) 63.06% had serum IL-6 ≥ 1.53 pg/mL whereas 18.18% had serum IL-6 < 1.53 pg/mL (*P* < 0.00001).

The results of univariable and multivariable logistic regression analysis and stepwise analysis of serum IL-23 levels in SpA patients with adjustment to CRP, BASDAI, and DMARDs showed no association. The adjusted OR for serum IL-23 ≥ 2.5 pg/mL in SpA patients treated with NSAIDs was 2.42 (95% CI 1.15–5.07) compared to patients not treated with NSAIDs (*P* = 0.02) (Table 3). The adjusted OR for serum IL-23 ≥ 2.5 pg/mL in AS patients was 2.07 (95% CI 1.06–4.057) compared to other SpA patients (*P* = 0.03) (Table 3).

## 5. Discussion

In this study, we evaluated the relationship between serum levels of IL-6 and IL-23 and activity of AS, PsA, and SAPHO syndrome. The role of IL-6 in SpA is so far not clearly demonstrated. Elevated levels of IL-6 have been described in patients with PsA compared with patients with psoriasis [19]. IL-6 was found to be increased in the serum and in the sacroiliac joints of patients with AS [1]. A correlation was found between serum levels of IL-6 and spinal inflammation as detected by magnetic resonance imaging (MRI) in AS [20]. A positive correlation has been reported between serum IL-6 and severity indexes such as those for vertebral mobility in AS [1]. A correlation was found between IL-6 level and BASDAI in AS [20]. Serum IL-6 levels were shown to be elevated in AS patients, along with factors associated with poor prognosis such as positive HLA-B27, inflammatory lower back pain, and arthritis [21]. A correlation between serum IL-6, CRP, and ESR in AS was shown [2]. Isolated cases of good treatment effect with tocilizumab in patients with AS and PsA have been reported [22–25].

On the other hand, data showing that IL-6 inhibition is not effective in SpA were reported [26, 27]. Treatment with tocilizumab was not effective in axial symptoms in AS patients, although it had the effect of reducing CRP [26]. A similar situation was described in PsA patients treated with tocilizumab; despite a complete normalisation of serum CRP, there was no improvement in either the joint or skin disease [27]. The role of IL-6 was not considered crucial in an animal model of tumour necrosis factor- (TNF-) mediated bilateral sacroiliitis [3].

In our study, we have shown increased serum IL-6 compared with control and found a positive correlation between serum IL-6 and disease activity measured by serum CRP and ESR in patients with AS, PsA, and SAPHO. This is in agreement with other publications [1, 2, 19]. In our opinion, this confirms the previously suggested pathogenic role of this cytokine in SpA. It will be necessary to conduct further studies on the possibility of using antibodies against IL-6 in selected patients with SpA.

IL-23 is a heterodimeric cytokine composed of two subunits, p40 (common with interleukin-12 (IL-12)) and p19. The relationship between IL-23 receptor polymorphism and the occurrence of AS and PsA has been described [28, 29]. A good clinical response to the use of antibodies against IL-23 in patients with PsA was also reported [30, 31]. All of this could have to confirm the role of IL-23 in SpA.

In previous small case studies, authors did not find a correlation between serum IL-23 and disease activity in AS and PsA patients [8, 9]. Wendling et al. [8] found no correlation between serum IL-12/IL-23 p40 and ESR, CRP, and BASDAI in 27 nonselected SpA patients. Melis et al. [9] showed a positive correlation between serum IL-23 levels and disease activity measured by CRP, ESR, and the number of swollen joints in patients with rheumatoid arthritis (RA), but they did not find such a correlation in 52 SpA patients (the group consisted of PsA and non-PsA spondyloarthritis patients). Histological changes of the synovial membrane correlated with the concentration of IL-23 in RA patients but did not in SpA patients. Furthermore, in RA patients they observed a reduction of serum IL-23 levels after treatment with TNF-alpha blockers, but this was not observed in patients with SpA [9]. The exacerbation of psoriasis induced by anti-TNF therapy has also been described in a patient with AS after switching to treatment with ustekinumab [32].

The results of our study, carried out on a larger group of SpA patients (152 patients), are in line with those described by other authors [8, 9]. We also did not observe a correlation between disease activity measured by ESR, CRP, and BASDAI and the serum concentration of IL-23 in SpA patients. There was also no difference between the serum concentration of IL-23 in the peripheral and axial forms of PsA and AS. However, SpA patients had significantly higher levels of IL-23 in comparison with the control group. Additionally AS patients had increased risk of increased serum IL-23 levels compared to other SpA patients. This could confirm suggestions about the role of this cytokine in the pathogenesis of SpA.

In situ analysis of IL-23-positive cells in the spine of patients with AS showed that IL-23 was expressed in the subchondral bone marrow and in fibrous tissue replacing bone marrow in facet joints of patients with AS [33]. The lack of relationship between serum IL-23 and disease activity in SpA can be explained by the fact that it acts locally in the joints and bones with no systemic effects [33]. It could confirm the previously suggested independent processes of ossification and inflammation in patients with AS. IL-23 might have a role in chronic changes in AS joints with no influence on laboratory disease activity. It will be necessary to conduct further studies on the possibility of using antibodies against IL-23 in AS patients. Perhaps the local rather than systemic application of IL-23 blockers had to make sense in the treatment of AS.

Despite the fact that, in our study, no correlation was found between serum IL-23 and disease activity in AS patients, this group of patients had an increased risk of elevated levels of IL-23.

Angiogenesis also plays a role in arthritis. Serum VEGF levels may be a marker of inflammatory activity in arthritis. In our previously published studies, we showed that serum VEGF levels correlated positively with disease activity assessed by CRP in PsA, but no significant correlations were found between levels of angiogenic cytokines and clinical presentation in SAPHO patients [34]. In our current study, we failed to show a correlation between serum IL-6 and IL-23 and the concentration of angiogenic cytokines in AS, PsA, and SAPHO.

The value of our work is the much larger number of SpA patients than that of previously reported studies. Moreover, no study has evaluated the relationship between serum IL-6 and IL-23 and angiogenic cytokines. The novelty of our work is that we assessed the association between IL-6, IL-23, and disease activity in patients with SAPHO syndrome. Little is known about the role of various cytokines in SAPHO. In the available literature, we did not find data concerning the role of IL-6 and IL-23 in SAPHO syndrome.

In summary, we emphasise that we have shown the relationship between serum IL-6 concentration and disease activity measured by ESR and CRP in AS, PsA, and SAPHO patients, while we have not found such a relationship in the case of serum IL-23. There was no correlation of serum IL-6 and IL-23 with clinical disease activity indexes in SpA patients.

The mechanism of action of IL-6 and IL-23 in patients with SpA requires further study to clarify their role in the pathogenesis of these diseases.

## Figures and Tables

**Table 1:** Clinical and laboratory characteristics of ankylosing spondylitis, psoriatic arthritis, and SAPHO syndrome patients and healthy controls.

Assessed parameter	Ankylosing spondylitis patients	Psoriatic arthritis patients	SAPHO syndrome patients	Healthy controls
	(*n* = 61)	(*n* = 69)	(*n* = 22)	(*n* = 29)
	Mean ± SD	Mean ± SD	Mean ± SD	Mean ± SD
	Median (Q1, Q3)	Median (Q1, Q3)	Median (Q1, Q3)	Median (Q1, Q3)
Age (years)	43.3 ± 13.2	52.0 ± 12.0	54.8 ± 12.3	48.2 ± 13.5
Sex	12 F. 49 M	39 F. 30 M	19 F. 3 M	19 F. 10 M
Disease duration (years)	10.0 (5.0, 18.0)	5.0 (2.0, 8.0)	2.0 (1.0, 4.0)	0
PASI	0.0	1.2 (0.0, 4.8)	—	0.0
BASDAI	5.72 (4.0, 7.8)	3.4 (2.3, 5.3)	3.6 (2.5, 5.0)	0
VAS pain (mm)	60.0 (40.0, 80.0)	40.0 (30.0, 60.0)	50.0 (40.0, 60.0)	0.0
CRP (mg/L)	9.93 (3.89, 17.1)	4.15 (1.85, 10.9)	4.2 (1.0, 10.6)	0.0
ESR (mm/h)	15.0 (7.0, 28.0)	13.0 (6.0, 22.0)	16.0 (6.0, 30.0)	9.4 (1.0, 26.0)
IL-23 (pg/mL)	0.3 (0.0, 2.8)	0.0 (0.0, 1.5)	0.0 (0.0, 0.3)	0.0 (0.0, 0.0)
IL-6 (pg/mL)	4.09 (1.8, 7.9)	2.83 (1.5, 6.2)	2.16 (1.0, 5.7)	1.2 (0.7, 1.5)
VEGF (pg/mL)	395.2 (220.0, 680.0)	291.4 (205.7, 652.7)	33.1 (222.5, 372.5)	300.1 (217.0, 437.6)
EGF (pg/mL)	120.0 (174.0, 186.0)	120.0 (60.0, 186.0)	126.0 (70.0, 208.0)	93.0 (45.0, 192.5)
FGFb (pg/mL)	0.0 (0.0, 0.0)	0.0 (0.0, 0.0)	0.0 (0.0, 0.0)	0.0 (0.0, 0.0)
FGFa (pg/mL)	0.0 (0.0, 0.0)	0.0 (0.0, 0.0)	0.0 (0.0, 24.3)	0.0 (0.0, 0.0)

Data are presented as number or mean ± standard deviation (Q1, Q3).

CRP: C-reactive protein.

EGF: epidermal growth factor.

ESR: erythrocyte sedimentation rate.

FGFa: acidic fibroblast growth factor.

FGFb: basic fibroblast growth factor.

IL-6: interleukin-6.

IL-23: interleukin-23.

*n*: number of patients.

PASI: Psoriasis Area and Severity Index.

SD: standard deviation.

VAS pain: visual analogue scale of patient's pain.

VEGF: vascular endothelial growth factor.

**Table 2:** A logistic regression model of the OR of the increased serum level of interleukin-6.

Covariates	Serum IL-6 ≥ 1.53 pg/mL		Serum IL-6 ≥ 6.64 pg/mL	
	OR (95% CI)	*P *	OR (95% CI)	*P *
Ankylosing spondylitis	2.04 (0.87–4.78)	0.10	1.30 (0.6–2.77)	0.51
Psoriatic arthritis	0.70 (0.32–1.51)	0.36	0.86 (0.40–1.84)	0.69
SAPHO	0.61 (0.23–1.65)	0.33	0.82 (0.28–2.41)	0.72
CRP ≥ 5 mg/L	7.68 (2.93–20.16)	<0.0001	15.76 (4.54–54.66)	<0.0001
BASDAI ≥ 4	2.90 (1.16–7.25)	0.02	1.11 (0.50–2.45)	0.79
NSAIDs	1.15 (0.42–3.11)	0.78	0.58 (0.20–1.65)	0.31
Sulfasalazine	0.64 (0.29–1.39)	0.26	1.07 (0.50–2.28)	0.85
Methotrexate	1.71 (0.71–4.15)	0.23	1.42 (0.65–3.11)	0.38

IL-6: interleukin-6.

OR: odds ratio.

95% CI: 95% confidence interval.

NSAIDs: nonsteroidal anti-inflammatory drugs.

**Table 3:** A logistic regression model of the OR of the increased serum level of interleukin-23 in spondyloarthritis patients.

Covariates	Serum IL-23 > 0.0 pg/mL		Serum IL-23 ≥ 2.5 pg/mL	
	OR (95% CI)	*P *	OR (95% CI)	*P *
Ankylosing spondylitis	1.83 (1.02–3.29)	0.04	2.07 (1.06–4.05)	0.03
Psoriatic arthritis	0.73 (0.41–1.31)	0.29	0.71 (0.36–1.40)	0.33
SAPHO	0.47 (0.18–1.26)	0.135	0.27 (0.06–1.21)	0.08
CRP ≥ 5 mg/L	1.02 (0.57–1.83)	0.94	0.93 (0.48–1.81)	0.82
BASDAI ≥ 4	1.52 (0.80–2.86)	0.19	1.47 (0.70–3.08)	0.30
NSAIDs	1.40 (0.70–2.80)	0.35	2.42 (1.15–5.07)	0.02
Sulfasalazine	1.08 (0.60–1.93)	0.79	0.58 (0.29–1.15)	0.12
Methotrexate	0.70 (0.37–1.30)	0.25	0.92 (0.45–1.86)	0.81

IL-23: interleukin-23.

OR: odds ratio.

95% CI: 95% confidence interval.

NSAIDs: nonsteroidal anti-inflammatory drugs.

## References

[1] Gratacós J., Collado A., Filella X. (1994). Serum cytokines (IL-6, TNF-alpha, IL-beta and IFN-gamma) in ankylosing spondylitis: a close correlation between serum IL-6 and disease activity and severity. *British Journal of Rheumatology*.

[2] Bal A., Unlu E., Bahar G., Aydog E., Eksioglu E., Yorgancioglu R. (2007). Comparison of serum IL-1*β*, sIL-2R, IL-6, and TNF-*α* levels with disease activity parameters in ankylosing spondylitis. *Clinical Rheumatology*.

[3] Hayer S., Niederreiter B., Nagelreiter I., Smolen J., Redlich K. (2010). Interleukin 6 is not a crucial regulator in an animal model of tumour necrosis factor-mediated bilateral sacroiliitis. *Annals of the Rheumatic Diseases*.

[4] Singh A. K., Misra R., Aggarwal A. (2011). Th-17 associated cytokines in patients with reactive arthritis/undifferentiated spondyloarthropathy. *Clinical Rheumatology*.

[5] Wendling D. (2008). Interleukin 23: a key cytokine in chronic inflammatory disease. *Joint Bone Spine*.

[6] Gaston J. S. H., Goodall J. C., Baeten D. (2011). Interleukin-23: a central cytokine in the pathogenesis of spondylarthritis. *Arthritis and Rheumatism*.

[7] Cauli A., Mathieu A. (2012). Th17 and interleukin 23 in the pathogenesis of psoriatic arthritis and spondyloarthritis. *Journal of Rheumatology*.

[8] Wendling D., Cedoz J.-P., Racadot E. (2009). Serum and synovial fluid levels of p40 IL12/23 in spondyloarthropathy patients. *Clinical Rheumatology*.

[9] Melis L., Vandooren B., Kruithof E. (2010). Systemic levels of IL-23 are strongly associated with disease activity in rheumatoid arthritis but not spondyloarthritis. *Annals of the Rheumatic Diseases*.

[10] Goldberger C., Dulak J., Duftner C., Weidinger F., Falkenbach A., Schirmer M. (2002). Vascular endothelial growth factor (VEGF) in ankylosing spondylitis—a pilot study. *Wiener Medizinische Wochenschrift*.

[11] Drouart M., Saas P., Billot M. (2003). High serum vascular endothelial growth factor correlates with disease activity of spondylarthropathies. *Clinical and Experimental Immunology*.

[12] Fink A. M., Cauza E., Hassfeld W. (2007). Vascular endothelial growth factor in patients with psoriatic arthritis. *Clinical and Experimental Rheumatology*.

[13] Butt C., Lim S., Greenwood C., Rahman P. (2007). VEGF, FGF1, FGF2 and EGF gene polymorphisms and psoriatic arthritis. *BMC Musculoskeletal Disorders*.

[14] van der Linden S., Valkenburg H. A., Cats A. (1984). Evaluation of diagnostic criteria for ankylosing spondylitis. A proposal for modification of the New York criteria. *Arthritis & Rheumatism*.

[15] Taylor W., Gladman D., Helliwell P., Marchesoni A., Mease P., Mielants H. (2006). Classification criteria for psoriatic arthritis: development of new criteria from a large international study. *Arthritis & Rheumatism*.

[16] Kahn M. F., Khan M. A., Wright V., Helliwell P. (1994). The SAPHO—syndrome. *Psoriatic Arthritis*.

[17] Ashcroft D. M., Li Wan Po A., Williams H. C., Griffiths C. E. M. (1999). Clinical measures of disease severity and outcome in psoriasis: a critical appraisal of their quality. *British Journal of Dermatology*.

[18] Garrett S., Jenkinson T., Kennedy L. G., Whitelock H., Gaisford P., Calin A. (1994). A new approach to defining disease status in ankylosing spondylitis: the bath ankylosing spondylitis disease activity index. *Journal of Rheumatology*.

[19] Alenius G.-M., Eriksson C., Dahlqvist S. R. (2009). Interleukin-6 and soluble interleukin-2 receptor alpha—markers of inflammation in patients with psoriatic arthritis?. *Clinical & Experimental Rheumatology*.

[20] Visvanathan S., Wagner C., Marini J. C. (2008). Inflammatory biomarkers, disease activity and spinal disease measures in patients with ankylosing spondylitis after treatment with infliximab. *Annals of the Rheumatic Diseases*.

[21] Londono J., Romero-Sanchez M. C., Torres V. G. (2012). The association between serum levels of potential biomarkers with the presence of factors related to the clinical activity and poor prognosis in spondyloarthritis. *Revista Brasileira de Reumatologia*.

[22] Brulhart L., Nissen M. J., Chevallier P., Gabay C. (2010). Tocilizumab in a patient with ankylosing spondylitis and Crohn's disease refractory to TNF antagonists. *Joint Bone Spine*.

[23] Shima Y., Tomita T., Ishii T. (2011). Tocilizumab, a humanized anti-interleukin-6 receptor antibody, ameliorated clinical symptoms and MRI findings of a patient with ankylosing spondylitis. *Modern Rheumatology*.

[24] Cohen J.-D., Ferreira R., Jorgensen C. (2011). Ankylosing spondylitis refractory to tumor necrosis factor blockade responds to tocilizumab. *Journal of Rheumatology*.

[25] Hughes M., Chinoy H. (2013). Successful use of tocilizumab in a patient with psoriatic arthritis. *Rheumatology*.

[26] Sieper J., Porter-Brown B., Thompson L., Harari O., Dougados M. (2014). Assessment of short-term symptomatic efficacy of tocilizumab in ankylosing spondylitis: results of randomised, placebo-controlled trials. *Annals of the Rheumatic Diseases*.

[27] Ogata A., Umegaki N., Katayama I., Kumanogoh A., Tanaka T. (2012). Psoriatic arthritis in two patients with an inadequate response to treatment with tocilizumab. *Joint Bone Spine*.

[28] Duan Z., Pan F., Zeng Z. (2012). Interleukin-23 receptor genetic polymorphisms and ankylosing spondylitis susceptibility: a meta-analysis. *Rheumatology International*.

[29] Rahman P., Inman R. D., Maksymowych W. P., Reeve J. P., Peddle L., Gladman D. D. (2009). Association of interleukin 23 receptor variants with psoriatic arthritis. *Journal of Rheumatology*.

[30] Gottlieb A., Menter A., Mendelsohn A. (2009). Ustekinumab, a human interleukin 12/23 monoclonal antibody, for psoriatic arthritis: randomised, double-blind, placebo-controlled, crossover trial. *The Lancet*.

[31] McInnes I. B., Kavanaugh A., Gottlieb A. B. (2013). Efficacy and safety of ustekinumab in patients with active psoriatic arthritis: 1 year results of the phase 3, multicentre, double-blind, placebo-controlled PSUMMIT 1 trial. *The Lancet*.

[32] Safa G., Martin A., Darrieux L. (2011). Exacerbation of infliximab-induced palmoplantar psoriasis under ustekinumab therapy in a patient with ankylosing spondylitis. *Journal of Clinical Rheumatology*.

[33] Appel H., Maier R., Bleil J. (2013). In situ analysis of interleukin-23- and interleukin-12-positive cells in the spine of patients with ankylosing spondylitis. *Arthritis and Rheumatism*.

[34] Przepiera-Będzak H., Fischer K., Brzosko M. (2013). Serum levels of angiogenic cytokines in psoriatic arthritis and SAPHO syndrome. *Polskie Archiwum Medycyny Wewnetrznej*.

